# Epicystic Surgical Fistula for Cystoscopic Exploration; Intravesical Treatment and Drainage

**Published:** 1889-06

**Authors:** John D. S. Davis

**Affiliations:** Birmingham, Alabama


### EPICYSTIC SURGICAL FISTULA FOR CYSTOSCOPIC EXPLORATION; INTRAVESICAL TREATMENT AND DRAINAGE.*

BY JOHN D. S. DAVIS, M. D., BIRMINGHAM, ALABAMA.

Epicystotomy has become an established and frequently practiced procedure, and the dangers incident to opening the bladder through the abdominal wall are so slight, that patients suffering from almost any vesical trouble are encouraged to have the bladder opened for diagnostic purposes and treatment, at a time when the general health remains unimpaired; a practice which, a few years ago, would not have been resorted to by the most aggressive surgeon.

Catarrh of the bladder, irrespective of its cause, is always followed by a series of consecutive pathological changes, which, independently of the partial or complete interruption of the passage of the urine, tend to destroy life. A dilatation of the bladder and ureters by retention of urine may give rise to such a degree of distension, as to destroy life from suspension of important functions by mechanical pressure. During the stage of inflammation a paretic condition may occur, the blood vessels in the vesical wall lose their support, and transudation and exudation take place into the paravascular tissue, which, combined with capillary stasis attending this stage of the disease, results in sloughing, infiltration, pyæmia, peritonitis and death. The damming up of the urine may, and does often, cause surgical-kidney, epididymitis and tetanus.

The treatment of chronic vesical catarrh resolves itself into a consideration of the causes producing the disease, many of which,

*Read before the State Medical Association of Alabama, April 11, 1889.

the presence in excess of certain inorganic constituents of the urine, stone, stricture and hypertrophy, are capable of correction; whilst others—such as malignant tumors and certain conditions of the prostate—may only admit of a palliation of the symptoms to which they give rise, and the removal of which must be the first object in treatment. But when a paretic condition of the bladder exists, provision must be made for the complete continuous emptying of the viscus; its thorough cleansing by frequent irrigation with hot, sterilized water; and the promotion of a healthy tone in the mucous membrane and muscular structure of the bladder. The frequent introduction of catheters for drawing off residual urine and washing out the bladder has been productive of much harm; and, instead of giving relief, proved to be, by reason of their frequent introduction into the inflamed bladder to draw off the urine two or three times a day, a source of immediate and alarming symptoms. These facts are cogent reasons for adopting surgical means in all cases of intravesical troubles as soon as a diagnosis can be made, and often when it cannot otherwise be made, for the complete emptying of the bladder, thorough cleansing, diagnosis, and intravesical treatment.

The epicystic surgical fistula is designed for drainage, intravesical treatment and cystoscopic exploration, and may be divided for consideration under the following heads:

I. Definition of epicystic surgical fistula.

II. Surgical resources in the formation of the epicystic surgical fistula.

1. Preparation for the operation.
2. Anæsthesia.
3. Position.
4. Incision and opening bladder.
5. Intravesical exploration and treatment.
6. Toilette and after-treatment.

III. Advantages of the epicystic surgical fistula.

1. Cystoscopic exploration.
2. Intravesical treatment.
3. Drainage.

### I.—DEFINITION OF EPICYSTIC SURGICAL FISTULA.

Epicystic Surgical Fistula is the title here given to a supra-pubic fistula into the bladder, created by the surgeon for exploration, intravesical treatment and drainage. A fistula, which, acting as an artificial urethra, is capable of giving free access to the inside of the bladder for cystoscopic exploration, to provide a ready, convenient and comfortable means of emptying the bladder at will, and gives the surgeon a competent opening into the viscus for intravesical applications.

It constitutes an essential element in the speedy and complete evacuation of the contents of the bladder in all epicystic operations, and imitates nature in the restoration of its own continuity and repair as the pathological changes within the bladder subside.

### II.—SURGICAL RESOURCES IN THE FORMATION OF THE EPICYSTIC SURGICAL FISTULA.

(1) *Preparation for the Operation.*—The presence of two assistants, though not necessary, may be of valuable aid. A temperature of 80° or 85° fahr. should be maintained in the operating room from the beginning to the end of the operation. All hair is to be shaved from the pubis, and all the details of antiseptic surgery are to be carried out so far as cleaning the pubis and abdomen. The bladder is emptied and thoroughly washed with warm water. When the water returns clean, the bladder is slowly distended with warm sterilized water thrown into the bladder by means of a fountain syringe, with nozzle in urethra—a degree of pressure sufficient to distend the bladder to its utmost capacity—which can never be too great for the resistance of the bladder. It is better to fail in filling the bladder than to distend the bladder beyond the limit of competency. Indeed it is not necessary to fill the bladder to any degree of resistance. I have operated when the bladder was in an irritable condition and would not tolerate distention greater than the capacity of two ounces, and had no difficulty in avoiding the prevesical fold of peritoneum or finding the bladder. The water is secured in the bladder by tying the penis at the base with a rubber tube.

A colpeurynter is next to be well oiled and inserted into the rectum—the rectum having been previously emptied by enema—and filled with warm water. This distension brings the bladder into view above the pubis.

(2) *Anæsthesia.*—My preference for chloroform is the result of my own personal experience with it. It is not free from objections, as its depressing effect on the heart is well known. The operation usually occupies fifteen minutes; and, hence, its prolonged use would be unnecessary and uncalled for. The objections to ether are the suppression of the excretions and the frequency with which bronchitis is produced when administered to persons advanced in years. The best course to pursue, when the operation is prolonged, is to follow the use of chloroform by ether. The patient must be kept profoundly under the influence of the anæsthetic from the first incision until the superficial wound is closed.

(3) *Position.*—The patient is placed on the back on an ordinary operating table, with the legs extended as if in a position for perfect comfort and rest. Many surgeons claim advantages in the position recommended by Trendelenburg. Eigenbrodt emphasizes the fact * that the elevation of the pelvis in Trendelburg's position † helps the surgeon to avoid the prevesical peritoneal fold at the time of the incision of the bladder.

I have employed this posture for intravesical operation by means of the supra-pubic incision with no advantage over the ordinary flat-back position. With two openings in the bladder for a continuous stream of clear water, I have no trouble in illuminating every part of the bladder with the electric surgical light, and thus am enabled to examine the entire intravesical wall. Undoubtedly the position recommended by Trendelenburg possesses advantages, which to the author more than myself, make it highly ideal. As for myself I prefer and recommend the flat-back position.

(4) *Incision and opening bladder.*—A perpendicular incision three or four inches long is made in the median line above the

*L. c., p. 72. Cf. Lang, Med. News, Dec. 4, 1886.

† In Trendelenburg's position the patient's legs are held over the shoulders of an assistant, with body resting on an inclined table, much in the position which hogs are swung for spaying.

symphysis pubis. The recti muscles are separated to symphysis. If the pyramidales are in the way, the fibres should be cut. The transversalis fascia is divided on a grooved director from symphysis to within one inch of upper margin of superficial wound. Instead of following Guyon's manœuvre, I catch the bladder with a tenaculum on a line with the symphisis, through the prevesical fat, and cut through with a bladder knife into the bladder with one smooth, clean incision, to prevent undue disturbance of the cellulo-adipose tissue between the bladder and pubis, and avoid infiltration. I have never seen a case where it was necessary to put up the prevesical fat, and with it the peritoneal cul-de-sac. If the bladder is caught on a line with the symphysis and cut downwards, no fears need be had for the peritoneum. Cutting this prevesical fat prevents its after dropping down over the opening into the bladder, and acting as a valve to prevent easy escape of urine and causing infiltration. And, too, such a procedure gives a smooth incision throughout, and it is almost impossible to have infiltration, even when no drainage tube is left in the bladder, and the urine is left to flow out through the fistulous track and taken up by a layer of absorbent cotton. In making the incision into the bladder, no attention is to be paid to any vein or veins which are sometimes met with. If cut they will stop bleeding when the bladder is dropped back and the rectal bag removed. The operation is usually bloodless in the sense of hemorrhage. I have operated without the patient losing more than one drachm of blood.

(5) *Intravesical exploration and treatment.*—The finger is carried into the bladder and a thorough search made for any tumors, villous growths or foreign bodies. The bladder is now emptied and the rubber around penis untied, and the bladder well washed out with hot sterilized water. The bladder can now be examined with the cystoscope and surgeon's electric light. If tumors be found, if practicable they should be removed; villous growths and any foreign body found should be removed. If nothing is found in the bladder, the surgical fistula, in the absence of malignancy, will be all that is required to relieve the cystitis.

(6) *Toilette and after treatment.*—The bladder is allowed to drop back into the pelvis and the superficial wound so closed by two sutures (including the skin and superficial fascia only), in the lower portion of the incision and one in the upper portion of the incision, as to leave a fistulous track of equal size from bladder to juncture of upper third and middle third of the superficial incision. A large rubber catheter is now to be introduced into the bladder through the opening, and its distal extremity allowed to enter a urinal placed in the bed between the patient's thighs, or preferably at the patient's side. Professor F. Trendelenburg, director of the surgical clinic of the University of Bonn, proposed, for draining the bladder in supra-pubic lithotomy, the T-tube in latero-abdominal position, and open wound treatment as the simplest, safest and best. He makes an antiseptic dressing of iodoform gauze around the T-tube. There can be no real necessity for a tube of any kind to be introduced into the bladder for the purpose of conveying the urine from the bladder to prevent infiltration, irritation of superficial fascia and soiling of dressings.

If the urine is kept acid, by the administration of citric acid, or some other more palatable acid drink, no better antiseptic than the acid urine can be secured for the constant bath of the parts. It should be allowed to flow out through the wound and absorbed by a pad of absorbent cotton placed loosely over the wound, and removed as often as soiled by the overflowing urine. By this method of emptying the bladder, no possible small amount of urine can be impeded in its outflow, which is the case around and outside of the tube, when catheter or tube is left in for any length of time—a source of no little annoyance at times. This little collected or retained urine, around the outside of the tube alone, I have seen produce a hard chill and elevation of temperature, and become for the time an immediate, alarming and aggravating source of trouble. I never have seen the skin made sore or chafed by the outflowing urine in epicystotomy, or from its after escape through the surgical fistula.

The bladder should be washed out twice daily with hot sterilized water, by means of a fountain syringe, with its nozzle introduced into the urethra, the water escaping through the epicystic fistula and guided into a bed-pan under the patient. The superficial stitches are taken out at the end of a week, and intermittent catheterization by the fistula is then resorted to, for the sole purpose of training the fistula and prevent its rapid closure. It is not necessary to catheterize for the purpose solely of drawing off the urine. In one case I never drew the urine save for the purpose of analysis, but occasionally introduced a rubber bougie to prevent the closure of the fistula. The drainage by the fistula alone is admirable, and the fistula will be well formed in twenty or thirty days, competent to retain urine without dripping and to allow its escape in a good projecting stream at will. With no tearing of the tissues, and with a clean cut, the drainage is perfect and the dangers are *nil.*

### III. ADVANTAGES OF THE EPICYSTIC SURGICAL FISTULA.

(1) *Cystoscopic Exploration.*—Nitze has by means of the cystoscope been enabled to diagnosticate tumors of the bladder in nine cases in which rectal palpation, the sound and other means had furnished negative results. One of the great difficulties in the cystoscopic exploration of the bladder is the presence of pus, mucus, and sometimes blood, which renders it exceedingly difficult to maintain a translucency of the fluid used to distend the bladder. By means of a simple fountain syringe a constant current of clear water may be kept within the bladder, so essential to a complete observation of the trigonum Lieutaudi, the most interesting part of the viscus, the ureters ; and to examine any affection of that viscus. The fistula may be made for temporary purposes of cystoscopy by the Peterson-Guyon-Perier operation ; but I can see great advantages from a different operation, by Dr. Hunter McGuire, the object of which tends to detect as well as eliminate the trouble within the viscus ; and, too, in the final construction of a permanent fistula, gives an easy after-method of exploration, and makes a better artificial method by reason of its length and extension upwards of two to three inches. Diagnostic purposes are met by the possibility of immediate detection of all local conditions, such as tumors, calculi, foreign bodies, neoplasms, the collection of fluids from the ureters, etc.

(2) *Intravesical Treatment.*—Having by means of the epicystic exploration revealed the true nature of the intravesical trouble, the treatment resolves itself into the immediate necessities of the case. For instance, prostatectomy may be necessary, villous papilloma may be found and should be remedied ; pedunculated growths may be found which should be removed by the scissors or Paquelin's cautery, etc. In such cases the opening in the bladder, sufficient to introduce the finger, should be enlarged downwards under the symphysis pubis and the operation indicated should at once be performed. The object of the formation of the permanent surgical fistula is to meet the after indications in such operations, the details of which do not properly come within the province of this discussion. However, it is sufficient to state, what is reasonable and practicable, that a better means by which the intravesical wall can be reached and treated therapeutically has not yet been devised.

(3) *Drainage.*—Permanent after-drainage in all intravesical operations cannot be necessary ; but is highly essential to secure good and sufficient drainage until the paravascular tissue is disengorged, the cystitis is relieved and the urine becomes normal and passes per urethra unobstructed. And until this end is attained complete artificial arrangement for the escape of the contents of the viscus must be made. In such cases of prostatic hypertrophy or malignant growths when removal of the obstruction is impossible or contraindicated, the epicystic surgical fistula is clearly indicated and essentially necessary. It meets every possible indication for local treatment and gives the only controllable, ready and free drainage to viscus and kidneys. Urinary back pressure as the result of incompetency of the urethra from the various immovable prostatic troubles, is often an immediate and remote cause of surgical-kidney, which can only be removed or relieved by supra-pubic drainage. In conditions of the bladder, of long standing cystitis as in the case reported by me in the Virginia Medical Monthly,* in which the urethra, though made com-

*Virginia Medical Monthly, April, 1889.
Alabama Medical and Surgical Age, April, 1889.
New York Medical Journal, April 13, 1889.

petent by cutting, was not sufficient to keep the bladder emptied without catheterization—a procedure which kept up a constant vesical inflammation, which, combined with capillary stasis attending the inflammatory process resulted in paresis.

I now have the pleasure of introducing that case, Mr. T. A. Nixon, to you fifty-eight days after the operation. His condition to-day is sufficient guarantee for all I have said in favoring the formation of an epicystic surgical fistula for the relief of chronic vesical catarrh. The result in this case is more than I promised. He can retain his urine several hours and without dripping of urine or pain to bladder. Urine completely under control and bladder relieved of pain. Present capacity of the bladder is twelve ounces. The fistula here is well formed and competent in every respect for which it was formed. I now draw his urine with a catheter, not that it is necessary to introduce the catheter, but to guide the urine into this bottle, and prevent a projecting stream from going on the floor.

